# Long Term *Ex Vivo* Culture and Live Imaging of *Drosophila* Larval Imaginal Discs

**DOI:** 10.1371/journal.pone.0163744

**Published:** 2016-09-29

**Authors:** Chia-Kang Tsao, Hui-Yu Ku, Yuan-Ming Lee, Yu-Fen Huang, Yi Henry Sun

**Affiliations:** 1 Institute of Molecular Biology, Academia Sinica, Taipei, Taiwan; 2 Institute of Genomic Sciences, National Yang-Ming University, Taipei, Taiwan; University of Massachusetts Medical School, UNITED STATES

## Abstract

Continuous imaging of live tissues provides clear temporal sequence of biological events. The *Drosophila* imaginal discs have been popular experimental subjects for the study of a wide variety of biological phenomena, but long term culture that allows normal development has not been satisfactory. Here we report a culture method that can sustain normal development for 18 hours and allows live imaging. The method is validated in multiple discs and for cell proliferation, differentiation and migration. However, it does not support disc growth and cannot support cell proliferation for more than 7 to 12 hr. We monitored the cellular behavior of retinal basal glia in the developing eye disc and found that distinct glia type has distinct properties of proliferation and migration. The live imaging provided direct proof that wrapping glia differentiated from existing glia after migrating to the anterior front, and unexpectedly found that they undergo endoreplication before wrapping axons, and their nuclei migrate up and down along the axons. UV-induced specific labeling of a single carpet glia also showed that the two carpet glia membrane do not overlap and suggests a tiling or repulsion mechanism between the two cells. These findings demonstrated the usefulness of an *ex vivo* culture method and live imaging.

## Introduction

The larval imaginal discs of *Drosophila* have been popular experimental subjects for the study of a wide variety of biological phenomena, including cell proliferation, differentiation, apoptosis, competition, shape change, intercellular signaling, and compartmental boundary formation. These discs derive from invagination of the embryonic epithelium and become flattened to form discs composed of two epithelial layers, namely the peripodial epithelium (PE) and the disc proper (DP). The imaginal disc cells proliferate through larval stages and begin to differentiate at the end of larval stage and develop into most of the adult body structures during the pupal period. Because of their flat and simple two-layer structure, they are easy to observe. However, live imaging of the discs is difficult because of their location deep within the larval body. Although it is possible to image live larva and pupa [1–5], the resolution and time window for observations are limited. *Ex vivo* culture of discs has been attempted since the 1960s [6, 7]. Pupal disc can be cultured for up to 96 hrs [8], and late larval discs can be induced to evaginate to mimic event in pupal development [9]. However, the long term *ex vivo* culture condition that allows normal development and cellular behavior of larval imaginal discs before evagination, when the disc is relatively flat and can be studied as a two dimensional system, has not been satisfactorily achieved.

The most challenging problem is finding a culture medium that will support the growth and development of the imaginal discs over a long period. Chemically defined medium were designed to mimic larval hemolymph composition [7, 10–12]. Using cells derived from imaginal discs to test for culture conditions, it was found that that addition of insulin, ecdysterone (20-hydroxyecdysone, 20HE), fly extract and fetal bovine serum (FBS) improved the cell survival and proliferation [11, 13]. Subsequent works on disc culture varied the concentration of these components [14–20] (summarized in S1 Table). Only up to 5–8 hours of culture was obtained [18, 19], although prepupal wing disc has been cultured for 10 hr to study disc eversion [16]. Late third instar wing disc has been cultured for 24 hr and allowed neuronal differentiation [21]. However, the condition has not been used by others and did not work for eye-antennal disc in our hands. We report that we have identified a culture condition that can sustain normal cell differentiation, morphogenesis and migration of imaginal discs for 18 hrs.

## Results and Discussions

### Long term culture of eye-antennal and wing discs

**Fig 1:**
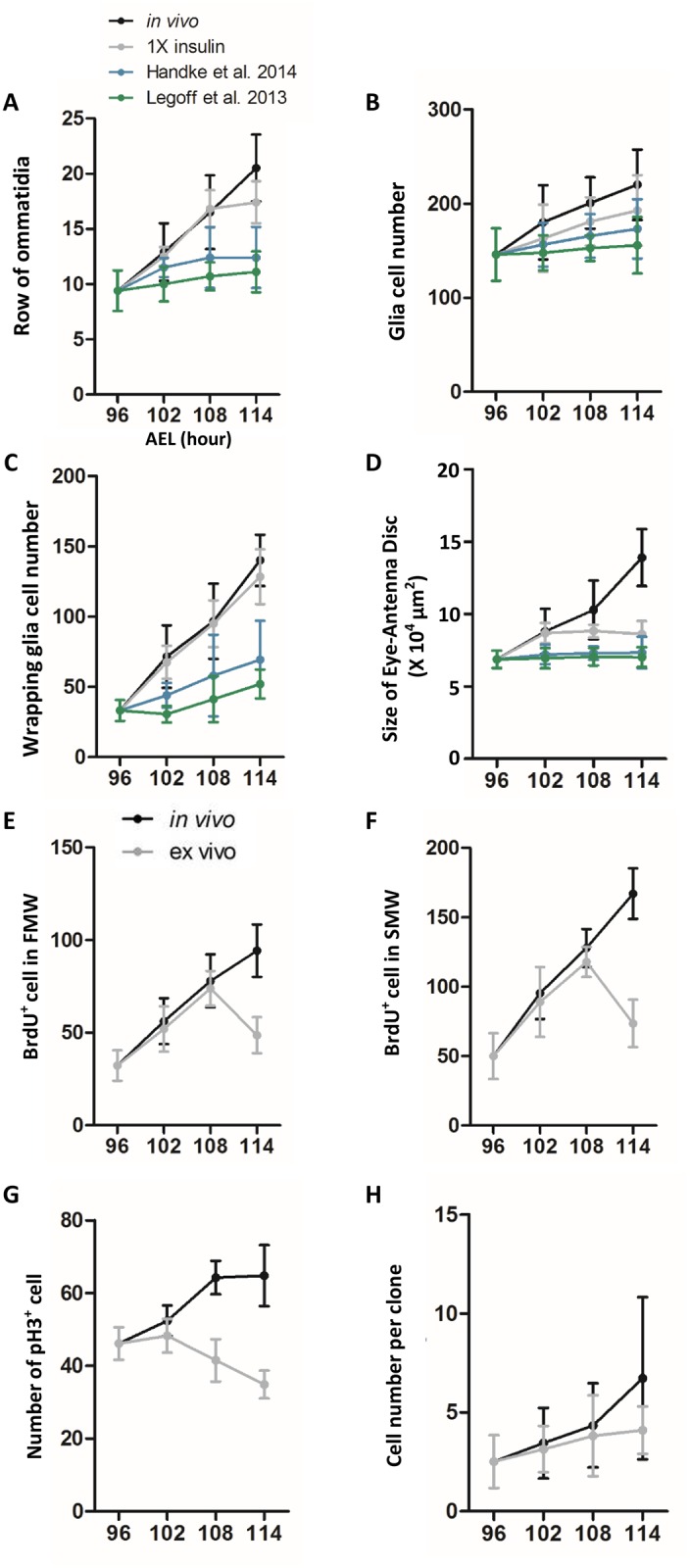
An improved medium for long-term *ex vivo* culture of eye-antenna disc. (A-D) Mid-third instar (96 hr AEL) *MZ97>RFP* eye-antennal discs were cultured *ex vivo* for 6, 12, and 18 hr (102, 108 and 114 hr AEL, respectively), and compared with discs dissected from larva (*in vivo*) at these time points. The discs were cultured in 1X insulin medium (this study) and the medium of Handke et al. (2014) and Legoff et al. (2013) (see S1 Table) respectively, for comparison. (A) The number of rows of ommatidia, marked by anti-HRP. (B) The number of retinal basal glia (marked by anti-Repo). (C) The number of wrapping glia, marked by *MZ97>RFP*. (D) The size of eye-antenna disc. (E-G) Mid-third instar (96 hr AEL) *w*^*1118*^ eye-antennal discs were cultured *ex vivo* for 6, 12 and 18 hr (102, 108 and 114 hr AEL, respectively), and compared with discs dissected from larva (*in vivo*) at these time points. (E) The number of BrdU^+^ cells in the first mitotic wave (FMW). (F) The number of BrdU^+^ cells in the second mitotic wave (SMW). (G) The number of phosphor-histone 3 (pH3^+^) cells in the entire disc. For all experiments, N = 10. (H) Neutral clones marked by nuclear GFP were induced at 72h AEL and the discs were dissected out at 96h AEL and cultured for 6, 12 and 18 hr (102, 108 and 114 hr AEL, respectively) and compared with clones in in freshly dissected disc. The number of cells in each clone were compared. N = 20.

Based on the effect on cell differentiation, migration and proliferation, rather than growth, we found a recipe of culture medium (S1 Table) that can sustain normal disc development for at least 18 hours. We cultured early third instar (84h AEL; S1 Fig) and mid-third instar (96h AEL; Fig 1) eye-antennal disc in the medium, without imaging, for various length and then stained for neuronal and glia markers. In terms of the number of rows of photoreceptor clusters (ommatidia) and the number of retinal basal glia (RBG) that migrated from optic stalk into the eye disc, and the number of wrapping glia (WG) as an indicator of glia differentiation, the *ex vivo* disc developed comparable to the *in vivo* discs at 6, 12 and 18 hr, except that the number of ommatidia rows was slightly reduced at 18 hr (Fig 1A–1C). The development slowed down at 24 and 36 hrs (S1A and S1B Fig). The progression of the morphogenetic furrow (MF) suggested that normal cellular morphogenesis can be supported. In 36 hr *ex vivo* cultured disc, the mature photoreceptor clusters were adjacent to the MF (not shown), suggesting that MF progression has stopped. For the first 12 hr in culture, the ommatidia row increased at a rate of 1.8 hr per row (Fig 1 and S1 Fig), in line with previous reports of 1.5 hr [22, 23] to 2 hr [24, 25] per row. The size of the ex vivo cultured disc was comparable to in vivo disc at 6 hr but did not increase thereafter (Fig 1D, S1C, S1F and S1I Fig). The number of S phase (BrdU^+^) cells in the first mitotic wave (FMW) and second mitotic wave (SMW) in the ex vivo cultured eye disc was comparable to the in vivo disc at 6 and 12 hr, but dropped significantly at 18 hr (Fig 1E and 1F). The number of mitotic (pH3^+^) cells in the entire disc was relatively normal at 6 hr, but become significantly reduced at 12 and 18 hr (Fig 1G). We also generated neutral clones marked by nuclear GFP at 72h AEL and dissected out the discs at 96h AEL for culture. The average cell number per clone in *ex vivo* cultured disc is comparable with *in vivo* disc up to 12 h in culture, and become significantly reduced at 18 h in culture (114h AEL) (Fig 1H). The cell density in ex vivo cultured eye disc did not differ significantly from the in vivo disc for 12 hr, but was reduced at 18 hr (S2 Fig). The cell density in ex vivo and in vivo antenna disc did not differ significantly for 18 hr (S2 Fig). The ex vivo cultured eye-antenna disc showed an increase in caspase 3 positive cells at 12 and 18 hr (S2 Fig), therefore probably offsetting the increase of cell numbers by cell divisions.

**Fig 2:**
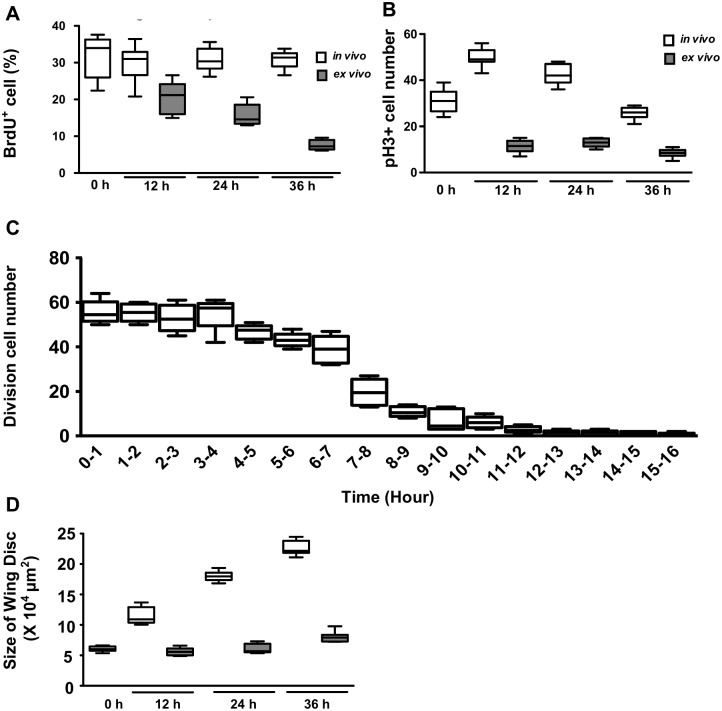
Long-term *ex vivo* culture of wing disc. Wing disc dissected from 84 h AEL larva (mid-L3) was cultured *ex vivo* for 12, 24 and 36 hr, respectively, and compared to disc directly dissected from larva (*in vivo*) at the corresponding time points. The discs were stained for BrdU incorporation and DAPI. (A) The number of BrdU^+^ cells gradually reduced in ex vivo cultured disc. N = 10. The percentage of S phase cells is consistent with previous report [46]. (B) The number of mitotic cells (pH3^+^) cells maintained at a low level throughout the 36 hr culture period. N = 10. (C) The number of cell divisions within each hour is monitored over 16 hr culture period of His2Av-GFP wing disc. N = 6 discs. Total cells = 2389. (D) The disc size (area) showed only slight growth over the 36 hr culture period.

In the wing disc cultured since early third instar (84 hr AEL), the number of S phase cells showed a reduction at 12 hr, and steadily decreased at 24 and 36 hr (Fig 2A). The number of mitotic cells showed a very significant reduction at 12 hr and remained low at 24 and 36 hr (Fig 2B). Live imaging of *ex vivo* cultured wing disc over 16 hrs showed a significant decrease in mitotic events after 7 hr (Fig 2C). After 12 hr, there is no more mitosis. The size of wing disc showed very little growth during the 36 hr *ex vivo* culture period (Fig 2D). In addition, the *ex vivo* cultured wing disc begin to show morphological changes at 48 hr in culture (data not shown), probably reflecting precocious disc eversion. The cell density is not significantly different between in vivo and ex vivo wing discs for at least 24 hr (S3E Fig). Caspase 3-positive cells in ex vivo disc was comparable to the in vivo disc for 24 hr and was higher only at 36 hr (S3D Fig).

In summary, our new medium can support the photoreceptor and glia differentiation in eye disc for 18 hr, sustained cell proliferation for 7 hr in wing disc and 12 hr in eye-antenna disc, but did not support growth in wing and eye-antenna discs.

The primary difference in our recipe is the high concentration of insulin (1250 μg/ml). We checked whether the insulin concentration is optimal (S1 Fig). Without insulin, there is very little increase in photoreceptor, glia and disc size even in the first 12 hours. With different concentrations of insulin (S1A–S1C Fig), the 1x and 2x supported the most differentiation, in terms of photoreceptor and glia number, but had no benefit on disc size. The insulin probably mimics the activity of endogenous factor, perhaps the insulin-like peptides. Interestingly, the human insulin was slightly better than bovine insulin in promoting photoreceptor differentiation (S1J and S1K Fig). We also found that the addition of larva extract (S1D–S1F Fig) or adult extract (not shown) did not have significant effect on disc differentiation and growth. Addition of various concentration of the moulting hormone 20HE reduced the development (S1G–S1I Fig). Addition of the insect blood sugar trehalose (60 μg/ml) at a level comparable to the hemolymph concentration was not beneficial (S1L and S1M Fig).

We compared our recipe with two other recent reports on culture method (S1 Table, Fig 1A–1D). Discs grown in our medium showed better development than those grown in the medium of Legoff et al. (2013) even at 6 h of culture, and better than those grown in the medium of Handke et al., (2014) at 12 hr. The differences are further enhanced at 18 hr. All three media do not support disc growth.

### Long term live imaging of wing disc

**Fig 3:**
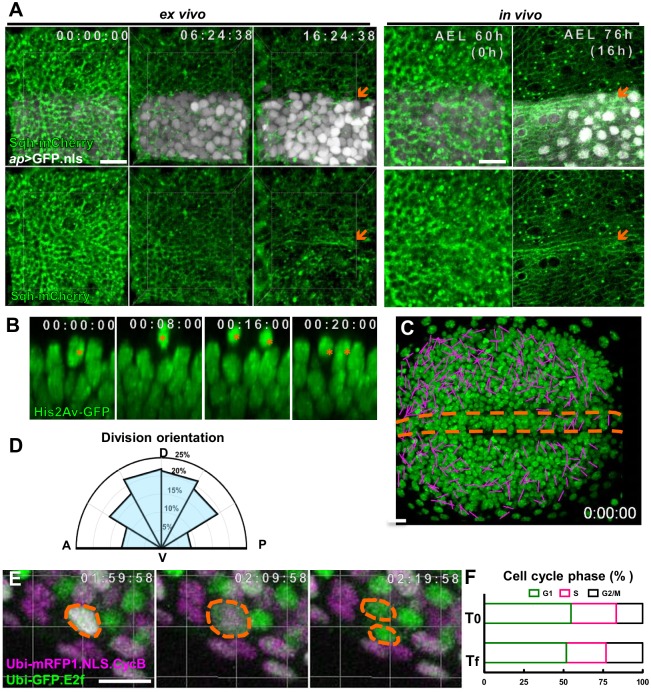
Cell proliferation and DV boundary formation in cultured wing discs. (A) Frames from S1 Movie of mid-L2 wing disc cultured *ex vivo* at 25°C for 16.5 hr (equals to early-mid-L3) compared with *in vivo* disc at the same age. *Sqh*-mCherry is Spaghetti squash (Sqh), a non-muscle myosin light chain, fused with mCherry [47]. Sqh-mCherry (green) showed the actomyosin cable at the DV boundary (arrow) at 16.5 hr. *ap-Gal4* driven nuclear GFP (*ap>GFP*.*nls*) (white) marked the dorsal compartment. The expression intensity gradually increased with time. N = 4 discs for *ex vivo*, N = 6 for *in vivo*. The Sqh-mCherry intensity in the dorsal (ap-GAL4 expressing domain) and ventral domain are not significantly different (ratio = 1.13±0.12, N = 6). The lack of nuclear signal in the left side of the image is due to disc curvature. (B-D) Frames and analyses based on S2 Movie of mid-L3 His2Av-GFP wing disc cultured for 16 hr. N = 4 discs. (B) XZ sections from S2 Movie, with apical surface on top. The dividing nuclei is marked by red asterisk. (C) The orientation of each cell division over the 7 hr period is marked by a line connecting the two daughter cells. (D) The orientation of cell divisions (based on C) showed a bias for divisions along the DV axis and against along the AP axis. (E, F) Frames and analysis based on S3 Movie. An early-L3 *ubi>Fucci* eye-antennal disc was cultured and monitored at 25°C for 14.5 hr. The fluorescent marker GFP-E2F_11–230_ (green) labels G2, M, and G1 phase, and mRFP1-CycB_1–266_ (magenta) labels the S, G2 and M phase. Their combination allows clear distinction of G1 (GFP only; green), S (RFP only; magenta), and G2 (GFP and RFP; white) phases. (E) A cell undergoes cell division, going from G2 (GFP and RFP) to two G1 (GFP) cells. (F) The proportion of different cell cycle phase at the start and end of the culture period are compared. N = 3 discs. In this and subsequent figures, the scale bar is 30 μm, except in (D, E) is 10 μm. This and subsequent analyses based on live imaging are based on at least three independent movies. The results were similar.

Wing disc from mid-second (mid-L2) instar larvae were cultured and monitored for 16.5 hr (at 25°C, equals to early-mid third instar; S1 Movie). 2% agarose cooled to room temperature has been used to embed the discs [16]. We used low-gelling-temperature agarose. This was effective in preventing the disc from drifting out of focal plane during imaging and minimize damage to the disc. Sqh-mCherry showed the actomyosin cable formed at the DV boundary (Fig 3A, arrow) at around 16.5 hr, corresponding to early L3. The timing is consistent with previous reports that the formation of actomyosin cable at the DV boundary occurs in early-L3 [26]. This result shows that the *ex vivo* cultured disc can develop from mid-L2 to early-L3, in a time comparable to *in vivo* development. It also shows that the pulse of the molting hormone 20HE at larval molt is not required for wing disc development from L2 to L3.

Cell divisions of cultured wing disc were analyzed by His2Av-GFP, which marks chromosomes. The mid-L3 wing disc was cultured and monitored for 7 hr (S2 Movie). Within this period, the cell density within the imaged area did not increase significantly. From 573 cells at time 0 in the imaged area, a total of 120 cell divisions were counted within the 7 hr period. The orientation of cell divisions was marked and showed a preference for the distal-proximal (DV) axis (Fig 3B and 3C), consistent with previous report [27, 28]. Cell division is relatively rare in the DV boundary region (Fig 3B), consistent with previous report [29]. The dividing nuclei ascended from the epithelium to the surface, divided and then sank into the epithelium again (Fig 3D), consistent with previous report [30].

Cell divisions were also monitored by the Fly-Fucci [31]. The fluorescent marker GFP-E2F_11–230_ labels G2, M, and G1 phases, and mRFP1-CycB_1–266_ labels the S, G2 and M phases. Their combination allows clear distinction of G1 (GFP only), S (RFP only), and G2/M (GFP and RFP) phases. For an early-L3 stage *ubi>Fucci* antennal disc monitored for 14.5 hr (S3 Movie), the cell number increased from 444 to 492. There were 39 mitotic events observed (a G2/M nuclei divided to generate two G1 nuclei; Fig 3E), accounting for most of the 48 new cells. The proportion of the cells in different cell cycle phases can be observed and showed no difference between the beginning and end of the 7 hr period (Fig 3F).

#### Long term live imaging of eye-antennal disc

**Fig 4:**
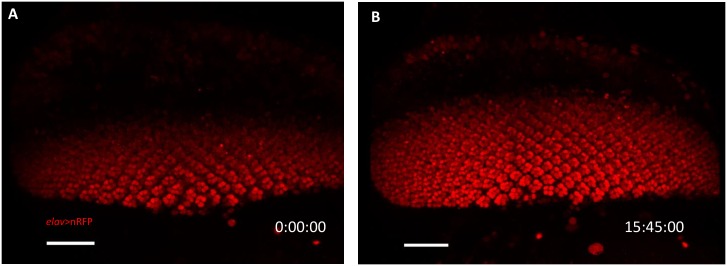
Ommatidial development in cultured eye-antennal disc. (A, B) Frames were taken from S4 Movie. A mid-L3 *elav>H2B-RFP* eye-antennal disc was cultured and monitored at 25°C for 15.75 hr. The RFP^+^ (Red) photoreceptor neurons appear posterior to the morphogenetic furrow (MF) and forms 8-cell ommatidial clusters. There are 6 rows of ommatidia in the beginning (A) and 11 rows at the end (B). N = 3 disc. The scale bar is 30 μm.

A mid-L3 *elav>H2B-RFP* eye disc was monitored for 15.75 hr (S4 Movie). During this period, the number of 8-cell ommatidial cluster increased from 65 to 226, and the number of rows of ommatidia increased at the rate of 3.15 hr/row (Fig 4), which is slower than the 1.8 hr/row calculated from disc freshly dissected at different time points (Fig 1). This is probably due to the effect on long term laser exposure. The nucleus can be seen to rise to the apical surface to divide and then descend basally, consistent with previous report [32]. Such divisions can be seen throughout the 15.75 hr culture and imaging period. The progression of MF can be seen in a mid-L3 Sqh-GFP eye-antennal disc cultured for 14.7 hr (S5 Movie).

**Fig 5:**
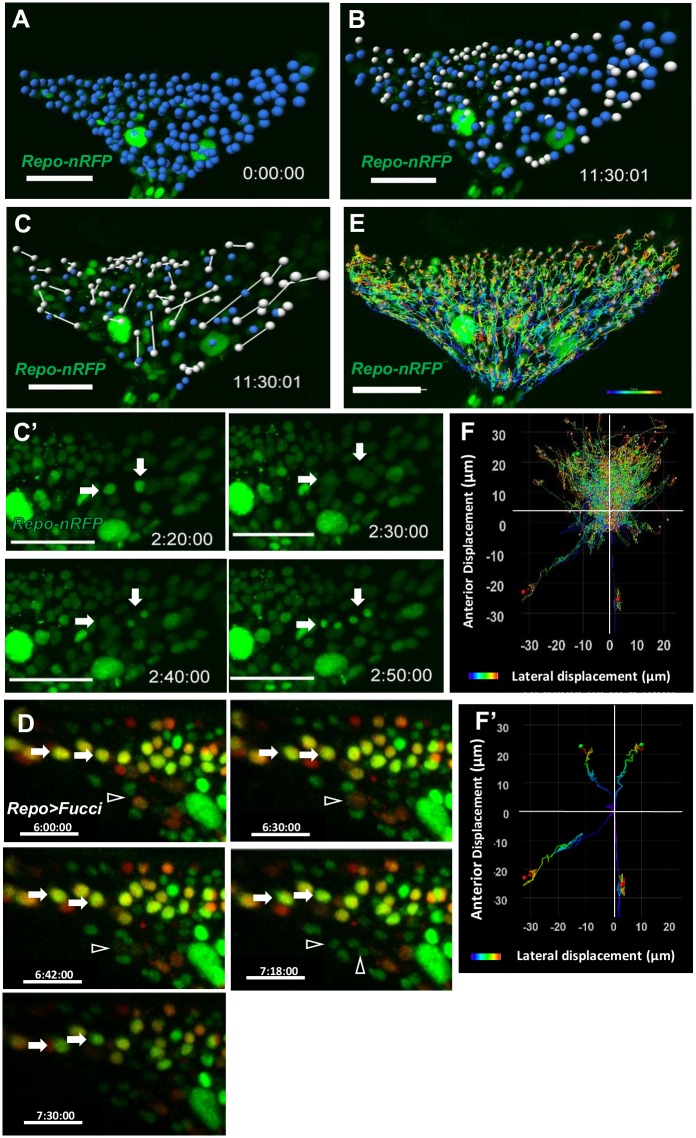
Proliferation and migration of retinal basal glia in cultured eye disc. Data were taken from S6 Movie. A mid-L3 *repo-RFP*.*nls* eye-antennal disc was cultured and monitored at 25°C for 11.5 hr, Nuclear RFP (green) marks the RBG nuclei. The Repo^+^ nuclei present at time 0 (A) are artificially marked in blue. The new Repo^+^ nuclei were marked in white (B). (C, C’) Division in two cells are shown (arrows). The two daughter cells of a division event are linked by a line. The sites of their divisions are marked by blue dots. (D) In *repo>Fucci* ex vivo cultured eye disc, a cell can undergo a full cell cycle (arrowhead showing progression from red (S phase) to no signal (mitosis phase) to two green (G1 phase) daughter cells). A cell can undergo endoreplication (arrows showing progression from yellow (G2 phase) to green (G1 phase) without undergoing mitosis). (E) The track of each Repo^+^ nucleus was recorded and color coded, with blue representing the start and red representing the end of movement. The two large Repo^+^ nuclei are the carpet glia. They do not undergo extensive movement. (F) The migration tracks are presented as displacements from their origin. (F’) The track of a few cells are presented. N = 3. The scale bar is 30 μm.

A group of glia (retinal basal glia, RBG), expressing the pan-glia marker Repo, migrate from the optic stalk into the eye disc [33, 34]. The anterior most of their distribution is 3–5 rows of cells behind the anteriorly progressing morphogenetic furrow (MF). Based on morphology and molecular markers, there are three major types of RBG, namely carpet glia (CG), surface glia (SG) and wrapping glia (WG) [35]. *repo-RFP*.*nls* allowed the live imaging of RBG behaviors (S6 Movie). Over a period of 11.5 hr, the number of Repo^+^ nuclei in a mid-L3 eye disc increased from 179 (Fig 5A; marked in blue) to 207 (Fig 5B; new Repo^+^ nuclei marked in white). 39 cell divisions were observed (Fig 5C, arrow points to dividing cells in C’), accounting for 92% of the 42 new RBG. The remaining 8% is due to migration from optic stalk. The two daughter cells of a division event are linked by a line (Fig 5C), and can disperse a long distance from the original site of division. *repo>Fucci* allowed the detection of glia cells undergoing a full cell cycle (Fig 5D, arrowhead), as well as cells that undergo endoreplication (Fig 5D, arrows).

The track of each Repo^+^ nucleus was recorded and color coded to show the start and end of movement (Fig 5E). The tracks are also presented as displacements from their origin (Fig 5F and 5F’). The tracks showed that most RBGs migrated toward the anterior of eye disc, but some migrate backwards, suggesting two distinct RBG populations with different migratory behaviors.

**Fig 6:**
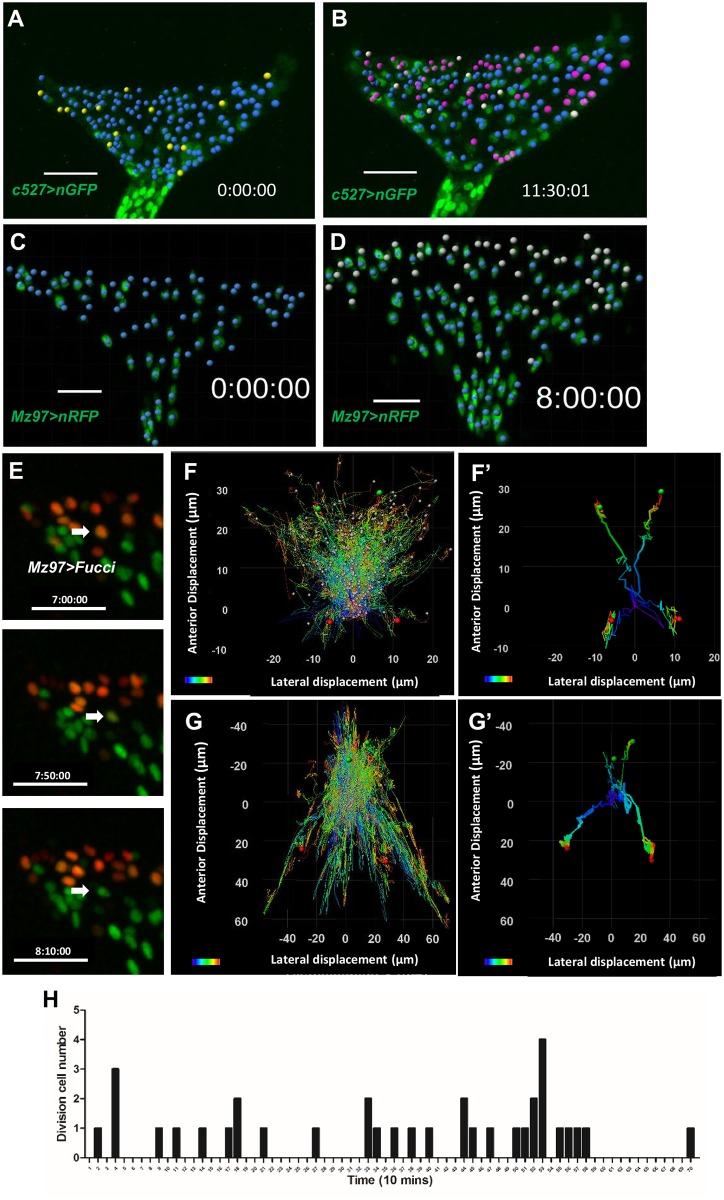
Proliferation and migration of surface glia and wrapping glia in cultured eye disc. (A, B, F) A *C527>H2B-RFP* mid-L3 eye disc was cultured and imaged for 11.5 hr (S7 Movie). (A) 152 C527^+^ nuclei were observed at the beginning. (B) 183 C527^+^ nuclei were observed at the end of 11.5 hr. Of the initial 152 cells, 110 did not divide (blue dots in A and B), 23 divided and both daughter cells are C527^+^ (purple dots), and 9 divided but only one daughter cell is C527^+^ (white dots). 14 C527^+^ nuclei (yellow dots in A) disappeared during this period. (C, D) A mid-L3 *MZ97>H2B-RFP* eye-antennal disc was cultured and monitored at 25°C for 20 hr (S8 Movie). (C) The MZ97^+^ nuclei present at time 0 are marked in blue. (D) The new MZ97^+^ nuclei are marked in white. No division was observed in MZ97^+^ nuclei. (E) *MZ97>Fucci* (S9 Movie) showed that the anterior MZ97^+^ cells (arrow) can progress from S phase (magenta) to G1 (green) without undergoing mitosis (magenta plus green). Only one round of endoreplication was observed for a WG during the observation period. (F, G) The migration tracks of C527^+^ (F) and MZ97^+^ (G) nuclei are presented as displacements from their origin, with blue representing the start and red representing the end of movement. (F’, G’) The isolated track of a few cells are shown as examples of the different behaviors. (H) Mitotic event in every 10 minutes in *C527>H2B-RFP* mid-L3 eye disc cultured for 11.5 hr (S7 Movie). The scale bar is 30 μm.

The *C527-Gal4* was used as a SG marker (Silies et al., 2007). A *C527>GFP*.*nls* mid-L3 eye disc was cultured and imaged for 11.5 hr (S8 Movie). In the beginning, there were 152 C527^+^ nuclei (Fig 6A). 110 of these did not divide (blue dots in Fig 6A and 6B). 23 divided and both daughter cells are C527^+^ (purple dots in Fig 6B). 9 divided but only one daughter cell is C527^+^ (white dots in Fig 6B). In total, 32/152 (21.0%) underwent division, similar to the 21.8% (39/179 in Fig 5) for Repo^+^ cells. Thus, the C527^+^ glia accounted for probably all proliferating RBG. 14 C527^+^ nuclei (yellow dots in Fig 6B) disappeared during this period, probably due to differentiation into another cell type and loss of the marker expression or by cell death. The occurrence of mitotic events was plotted every 10 minutes (Fig 6H). The divisions were scattered, but only one division occurred after 10 hr, suggesting that the division may not be sustained normally after 10 hr in culture. The migration displacement chart showed that the C527^+^ nuclei mostly migrated forward (Fig 6F and 6F’).

The WG were monitored by *MZ97>H2B-RFP* (S7 Movie). Over a period of 20 hr, the MZ97^+^ nuclei in a mid-L3 eye disc increased from 86 to 181. The 47 new MZ97^+^ nuclei are marked in white during 8 hr live imaging (Fig 6C and 6D). However, no division was observed in MZ97^+^ nuclei. This is consistent with previous reports that MZ97^+^ cells are post-mitotic, based on the finding that blocking cell division in WG did not reduce RBG cell number [35]. Live imaging showed that all the new MZ97^+^ nuclei appeared in the anterior front, rather than migrating from the optic stalk. This is also consistent with the sequential differentiation model that WG differentiates from migrating SG upon contact with photoreceptor axon at the anterior front [35]. Our live imaging provided direct evidence that MZ97^+^ cells differentiate *in situ* from Repo^+^ cells at the anterior front. Interestingly, *MZ97>Fucci* (Fig 6E and S9 Movie) showed that the anterior MZ97^+^ cells can progress from S phase (magenta) to G1 (green) without undergoing mitosis (magenta plus green), suggesting that MZ97 cells can undergo endoreplication, probably accounting for the endoreplication seen in *repo>Fucci* (Fig 5D). The migration tracks showed that most MZ97^+^ nuclei migrate toward the posterior (Fig 6G and 6G’).

The CGs are two giant glias with membrane extended to cover the entire region of RBG. *C135-Gal4*, a CG-specific Gal4, driven *moesin-GFP* (*C135>moesin-GFP*) showed membrane extension with dynamic protrusions in the anterior front (S10 Movie). The anterior front of CG membrane extended 21.7 μm during the 7.5 hr of imaging.

#### *Ex vivo* manipulations

**Fig 7:**
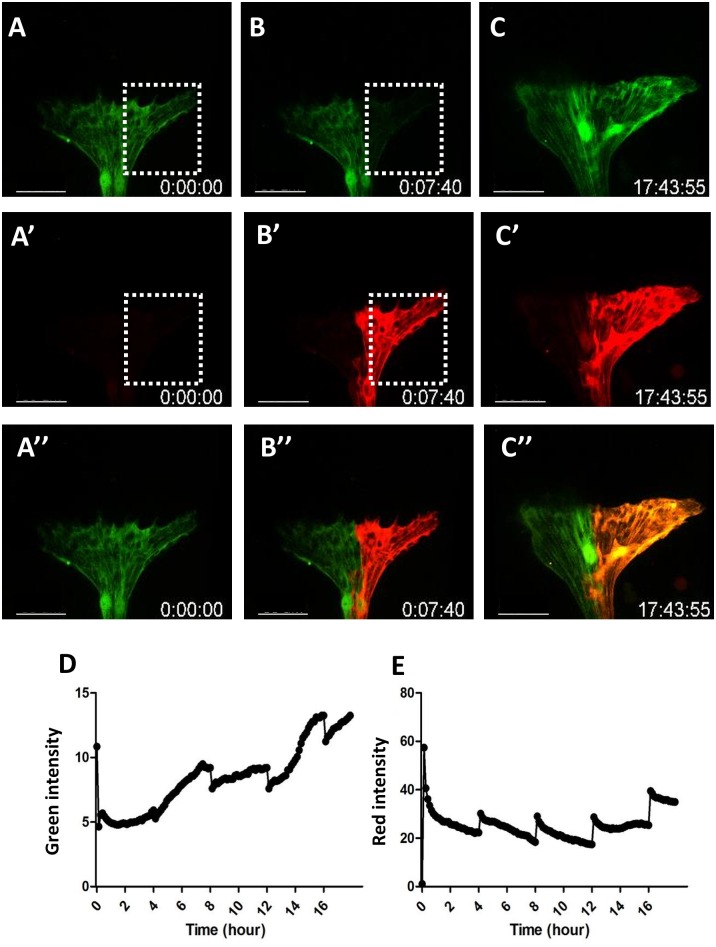
Selectively labeling of single carpet glia. (A-C) The KAEDE fluorescent protein was expressed in the carpet glia by the *C135-Gal4* driver. One of the two CGs was selectively illuminated by 514 nm laser in the boxed area. The KAEDE was photoconverted from green to red and the signal spread to the entire cell very rapidly. (D) The green signal showed nearly complete conversion to red and slowly recovered due to new protein synthesis. (E) The red signal decayed slowly, probably due to protein degradation. Subsequent boosts at lower laser power was at every 4 hr to maintain the red signal. N = 3. The scale bar is 50 μm.

With the ability of long term *ex vivo* culture, it is possible to selectively manipulate specific cells. For example, we specifically expressed the KAEDE fluorescent protein (Ando et al., 2002; Mizuno et al., 2003; Chen et al., 2012) in the CGs using the *C135-Gal4* driver. We then selectively labeled one of the two CGs by UV-converting the KAEDE from green to red (Fig 7). The converted signal spreads to the entire cell almost instantaneously. This cell-specific labeling allowed the visualization of the extent of the membrane of each of the two CGs. It showed almost no overlap between the two CGs, suggesting a tiling or repulsion mechanism between the two cells.

## Conclusions

In summary, our method allows the long term *ex vivo* culture and live imaging of the larval imaginal discs. We tested wing, antenna and eye discs, and demonstrated that although the culturing condition does not support disc growth and only sustained cell divisions for up to 7–12 hrs, the cultured discs undergo normal developmental process, such as cell proliferation, differentiation, migration and membrane dynamics for up to 18 hrs. The method provided opportunity for directly observing the temporal sequence of biological events, much better than deducing the sequential events by piecing together multiple snap shots at different time points and often from different samples. It allows the selective labeling or manipulation of specific cells with laser precision, replacing or supplementing genetic labeling. We used laser activated KAEDE to specifically label a single carpet glia and observed its entire membrane extension (Fig 6). We can also use laser to specifically disrupt the actomyosin network, by the chromophore-assisted laser inactivation (CALI;[36]) method, in the disc to examine the physical tension among cells (HYK and YHS, submitted). It also allows the easy test of chemicals by adding directly to the culture medium, thus maybe used as a platform for drug screens. Although we focused on imaginal discs, the condition may also be suitable for other larval tissues.

Our live imaging of the behaviors of RBGs in eye disc provided direct proof that the MZ97^+^ WG differentiate from existing Repo^+^ cells, and possibly from the C527^+^ SG. These results are consistent with the sequential differentiation model [35]. Our results further showed that SG and WG have distinct migratory behaviors and suggested that WG undergoes endoreplication. We also showed that the two giant carpet glia membrane do not overlap, suggesting a tiling or repulsion mechanism between the two cells.

## Materials and Methods

### Fly stocks

The fly stocks used are: *sqh*^*AX3*^ *Sqh-GFP* [37](provided by Jui-Chou Hsu), *Sqh-mCherry* [38] (provided by Yu-Chiun Wang), *UAS-H2B-RFP* [39], *UAS-KAEDE* (Chen et al., 2012) (provided by Ann-Shyn Chiang). *repo-RFP*.*nls* [40], *ap*^*md544*^*-Gal4* [41], *His2Av-EGFP* [42], *UAS-Fucci* and *ubi-Fucci* (*ubi-GFP-E2F*_*11-230*_ *ubi- mRFP1-NLS-CycB*_*1-266*_) [31], *UAS-Moesin-GFP* was from Drosophila Genetic Resource Center. *hs-FLP*^*122*^, *Ay-Gal4 UAS-GFP*, *C135-Gal4*, *elav-Gal4*, *C527-Gal4*, *MZ97-Gal4 and UAS-GFP*.*nls* were from Bloomington Drosophila Stock Center.

### Culture medium

Schneider’s Drosophila medium (Thermo 21720–024) was supplemented with 2% FBS (Sigma N4765), 0.5% penicillin-streptomycin (GIBCO0759), 0.08% fly extract and 1.25 mg/ml insulin (Sigma I9278). Prepared culture medium should be used within a month.

### Fly extract

About 0.35 g of larva or adult (about 300 each) were frozen in a 1.5 ml Eppendorf at -80°C for at least 45 minutes, and grind in 2.5 ml Schneider’s Drosophila medium with a disposable micro tissue homogenizer (Thomas No. 1215D61). The homogenate was centrifuged at 1500 Xg (KUBOTA 1720, rotor RA-48J) at 4°C for 30 minutes. The supernatant was decanted into a fresh Eppendorf tube and incubated at 60°C for 10 minutes to inactivate protease, centrifuged again at 1500 Xg at 4°C for 60 minutes. The supernatant was sterilized through a 0.22 μm filter. The fly extract (0.14 g/ml) is stored at 4°C and used within a week.

### Low melting agarose

0.75% low-gelling-temperature agarose (Agarose II, AMRESCO 0815-25G) in 1xPBS (10X PBS: 80 g NaCl, 14.4 g Na_2_HPO_4_, 2.4 g KH_2_PO_4_, 2 g KCl per 1 L MilliQ H_2_O, adjust to pH 7.0) was molten by microwave and kept at 37°C. It should be used within a month. The agarose was boiled every time after use to avoid contamination.

### Dissection of discs

All equipment (forceps, pipette, cover slide, etc.) were sterilized with 70% ethanol. The one eye disc, with hemi-brain and mouth hook attached, was dissected carefully in culture medium. The wing disc was dissected as detached from larval epidermis.

### Culture and live imaging

The culture chamber (PECON, POC-R2 Cell Cultivation System) components were soaked for at least 12 hours in 70% ethanol, and assembled on a 0.17 mm cover slide on the stage acceptor. The cover on the chamber was removed, and the disc sample was placed onto the cover slide using a pipette. The orientation of disc was adjusted by forceps so that the disc lies flat on the slide. Most of the medium was removed and 5–6 μl 0.75% low gelling agarose (37°C) was added to the sample. The melted gel was added vertically to force the eye disc remain at the bottom of the gel. After 5 minutes for the agarose to solidify, 1 ml culture medium at 25°C was added slowly to the chamber. The chamber was then loaded onto the Zeiss LSM 710 inverted confocal microscope in the incubator equipped with CTI controller at 25°C.

### Imaging of *ex vivo* larval disc

The *ex vivo* time-lapse images were acquired with Zeiss LSM 710 with a GaAsp detector or 510 META-NLO laser-scanning confocal microscope equipped with culture chamber maintained in 25°C and continuous support of humid air (25°C). C-Apochromat 40x/1.2W Korr or LD C-Apochromat 40x/1.1W objectives were used for most of the imaging. Z-stack images were acquired every 4, 10, or 15 minutes as indicated in the movies. 30–60 slices per stack were used based on the type of fluorescent proteins (nucleus/membrane) from different samples. Optical sections were set to 1.2 μm with optical interval less than 1 μm. Eye-antennal disc size and growth change were acquired using Plan-Apochromat 20x/0.8 objective. 45 slices were stacked under optical section set to 1.4 μm with optical interval equals to 0.8 μm. Time is displayed as hr:min:sec, relative to the start of the time-lapse.

### Clone induction

The clones were induced at 72h AEL for 7 mins heat shock, and dissected at 96h AEL.

### KAEDE photoconversion

The green KAEDE was excited by a 514 nm laser and the red 543 nm emission was monitored. The initial photoconversion was 3% power of a 25 mW laser for 80 scans. Each scan takes 1.4 sec to complete. Because the photo-conversion is due to a peptide cleavage [43], it is irreversible and stable. The red signal slowly decays, probably due to protein degradation. The green signal showed nearly complete conversion to red and slowly recovered due to new protein synthesis. Subsequent boosts were reduced to 1% of 25 mW laser for 40 scans of 1.4 sec each, totaling 1 min, every 4 hr to maintain the red signal.

### Immunostaining

Larval discs were dissected, fixed and stained followed by protocol as previous described [44]. Primary antibodies were: mouse-anti Cut (1:200), mouse anti-Repo (1:100), rat-anti-Elav (1:200), rat-anti E-Cadherin (1:25) from Developmental Studies Hybridoma Bank (DSHB, University of Iowa). The anti-cleaved caspase 3 (CC3) antibody [45] is from Cell Signaling Technology 9661S. Fluorescence conjugated secondary antibodies, including anti-HRP (1:300), were obtained from Jackson ImmunoResearch. Imaging procedures were acquired by LSM 510 Meta or LSM 780 confocal microscope (Zeiss).

## Supporting Information

S1 Table Comparison of medium compositions. In order for comparison, we standardized the compositions of various mediums reported for disc culture. FBS, fetal bovine serum; FCS, fetal calf serum. The serum is heat inactivated. The standard unit of antibiotics is 10 U/ml penicillin and 10 μg/ml streptomycin. The standard fly or larva extract is 0.14 g/ml in Schneider’s medium. We found that larval and adult extract are equivalent. We used recombinant human insulin, whereas bovine insulin purified from pancreas was used in other studies [13, 15, 18, 20]. The source for the other reports is not known. We have not tested whether the species source makes a difference. The concentration conversion for insulin in Zartman et al (2013) is 2.5 U/mg. Ecdysone is 20-hydroxyecdysone. # juvenile hormone analog and fat body conditioned medium. § 50 μg/ml penicillin, 50 μg/ml streptomycin, 100 μg/ml neomycin.
(DOCX)

S1 Fig Optimization of the culture medium. Early third instar (84 hr AEL) *w*^*1118*^ eye-antennal discs were cultured *ex vivo* for 12 hr, 24 hr and 36 hr (96, 108 and 120 hr AEL), respectively, and compared with discs freshly dissected from larva (*in vivo*) at these time points. (A-C) Different concentration of insulin were compared. (D-F) The 1X insulin medium with addition of different concentration of larva extract were compared. (G-I) The 1X insulin medium with addition of different concentration of 20HE were compared. (J, K) The insulin from human or bovine were compared. (L, M) The 1X insulin medium with addition of trehalose (60 μg/ml) was compared. (A, D, G, J, L) The number of rows of ommatidia, marked by anti-HRP. (B, E, H) The number of retinal basal glia (marked by anti-Repo). (C, F, I, K, M) the size of eye-antenna disc. In all experiments, N = 20.
(TIF)

S2 Fig Cell density and cell death in ex vivo cultured eye-antenna disc. The eye-antenna disc at 96h AEL stained with DAPI (blue). The two insets (green and red, from the antenna and eye disc, respectively) are enlarged as A’ and A”. (B-B”) The in vivo disc at 102h (B, +6), 108h (B’, +12) and 114h (B”, +18) AEL. (C-C”) The ex vivo disc at 102h (C, +6), 108h (C’, +12) and 114h (C”, +18) AEL. The discs were stained with DAPI (blue) and anti-cleaved caspase 3 (CC3, red). The cell density in the in vivo and ex vivo eye disc (D) and antenna disc (D’) were plotted for the 18 hr culture period. (E) The number of CC3^+^ cells in the in vivo and ex vivo eye-antenna disc were plotted for the 18 hr culture period. The Scale bar is 30 μm, except for A’ and A” (5 μm).
(TIF)

S3 Fig Cell density and cell death in ex vivo cultured wing disc. The wing disc at 84h AEL stained with DAPI (blue). The insets (green) is enlarged as A’. (B-B”) The in vivo disc at 96h (B, +6), 108h (B’, +12) and 120h (B”, +18) AEL. (C-C”) The ex vivo disc at 96h (C, +6), 108h (C’, +12) and 120h (C”, +18) AEL. The discs were stained with DAPI (blue) and anti-activated caspase 3 (CC3, red). The cell density in the in vivo and ex vivo wing disc (D) were plotted for the 18 hr culture period. (E) The number of CC3^+^ cells in the in vivo and ex vivo wing disc were plotted for the 18 hr culture period. The Scale bar is 30 μm, except for A’ (5 μm).
(TIF)

S1 Movie DV boundary formation in cultured wing disc. The mid-L2 wing disc, with associated trachea, was cultured for 16.5 hrs (about early-mid-L3). Sqh-mCherry (green) showed the appearance of the actomyosin cable at the DV boundary (arrow) at around 16 hr. *ap-Gal4* driven nuclear GFP (*ap>GFP*.*nls*) (white) marked the dorsal compartment. The expression intensity gradually increased. The scale bar is 30 μm.
(AVI)

S2 Movie Cell proliferation in cultured wing disc. The mid-L3 wing disc was cultured for 7 hr. His2Av-GFP (green) marked chromosomes. The two daughter cells of a division event are linked by a line. The scale bar is 30 μm.
(AVI)

S3 Movie Cell divisions in cultured antennal disc. An early-L3 *ubi>Fucci* eye-antennal disc was cultured and monitored at 25°C for 12 hr. The cell cycle G1 (GFP only; green), S (RFP only; magenta), and G2 (GFP and RFP; white) phases can be distinguished. The scale bar is 30 μm.
(AVI)

S4 Movie Ommatidial development in cultured eye disc. A mid-L3 *elav>H2B-RFP* eye-antennal disc was cultured and monitored at 25°C for 15.75 hr. RFP (Red) marks the differentiating photoreceptor neurons. The scale bar is 30 μm.
(AVI)

S5 Movie Progression of morphogenetic furrow in *ex vivo* cultured eye-antennal disc. An early-L3 *Sqh-GFP* eye-antennal disc was cultured and monitored at 25°C for 14.5 hr. Sqh-GFP (green) marks the cell contour. The scale bar is 30 μm.
(AVI)

S6 Movie Proliferation and migration of RBG in cultured eye disc. A mid-L3 *repo-nRFP* eye-antennal disc was cultured and monitored at 25°C for 11.5 hr, RFP (green) marks the RBGs. While most Repo^+^ nuclei move toward the anterior, some migrate backward. The two large nuclei are the carpet glia. The scale bar is 30 μm.
(AVI)

S7 Movie Proliferation and migration of C527^+^ glia in cultured eye disc. A *C527>GFP*.*nls* mid-L3 eye-antennal disc was cultured and imaged at 25°C for 11.5 hr. GFP (green) marks the SG. The scale bar is 30 μm.
(AVI)

S8 Movie Migration of MZ97^+^ glia in cultured eye disc. A mid-L3 *MZ97>H2B-RFP* eye-antennal disc was cultured and monitored at 25°C for 20 hr. RFP (green) marks the WG. Note the new MZ97^+^ nuclei appear at the anterior front, and many nuclei move backward. The scale bar is 30 μm.
(AVI)

S9 Movie MZ97^+^ glia undergoes endoreplication. A mid-L3 *MZ97>Fucci* eye-antennal disc was cultured and monitored at 25°C for 23 hr. The anterior MZ97^+^ cells can progress from S phase (magenta) to G1 (green) without undergoing mitosis (magenta plus green). The scale bar is 30 μm.
(AVI)

S10 Movie Membrane dynamics of carpet glia in cultured eye disc. A mid-L3 *C135>Moesin-GFP* eye-antennal disc was cultured and monitored at 25°C for 7.5 hr. Moesin-GFP (green), Repo (magenta). The scale bar is 30 μm.
(AVI)

S11 Movie Selectively labeling of a single carpet glia. A mid-L3 *C135>KAEDE* eye disc was cultured and monitored at 25°C for 17.75 hr. The photoconversion was induced by 405nm laser. Immediately after the two minutes of laser scan for photoconversion, the red signal is seen in the entirety of the carpet glia. The scale bar is 50 μm.
(AVI)
