# Supplementary material for: Long Term Ex Vivo Culture and Live Imaging of Drosophila Larval Imaginal Discs
Source: PLoS One. 2016 Sep 29;11(9):e0163744. doi: 10.1371/journal.pone.0163744 (PMC5042436; doi:10.1371/journal.pone.0163744)
Supplement: S1 Table — In order for comparison, we standardized the compositions of various mediums reported for disc culture. FBS, fetal bovine serum; FCS, fetal calf serum. The serum is heat inactivated. The standard unit of antibiotics is 10 U/ml penicillin and 10 μg/ml streptomycin. The standard fly or larva extract is 0.14 g/ml in Schneider’s medium. We found that larval and adult extract are equivalent. We used recombinant human insulin, whereas bovine insulin purified from pancreas was used in other studies [13, 15, 18, 20]. The source for the other reports is not known. We have not tested whether the species source makes a difference. The concentration conversion for insulin in Zartman et al (2013) is 2.5 U/mg. Ecdysone is 20-hydroxyecdysone. # juvenile hormone analog and fat body conditioned medium. § 50 μg/ml penicillin, 50 μg/ml streptomycin, 100 μg/ml neomycin. (DOCX) [file pone.0163744.s001.docx]

| Reference | Medium | Serum | Antibiotics | Fly Extract | Insulin | Ecdysterone | Use |
| --- | --- | --- | --- | --- | --- | --- | --- |
| Davis & Shearn, 1977 | X (XCS) |  |  | ＃ | 0.4 mU/ml | 1 ng/ml | discs |
| Wyss, 1982 | ZW |  |  | 22.5% | 10 μg/ml | 10 ng/ml | disc cells |
| Currie et al., 1988 | Shields and Sang M3 | 2% FBS |  | 5% | 125 mU/ml | 1 ng/ml | disc cells |
| Schubiger & Truman, 2000 | Shields and Sang M3  D22 | 7.5% FCS | § |  |  | 1 μg/ml | wing disc, 24 hr |
| Gibson et al., 2006 | Shields and Sang M3 | 10% FBS | 1X |  | 0.01 mU/ml |  | 1.5-2 hr |
| Cafferty et al., 2009 | Schneider’s | 1% FBS | 10X |  | 200 μg/ml |  | 4 hr |
| Aldaz et al., 2010 | Shields and Sang M3 | 2% FCS | 5X |  |  | 100-500 ng/ml | disc eversion |
| Ohsawa et al., 2012 | Schneider’s | 10% FBS |  |  |  |  | 3 hr |
| Zartman et al., 2013 | Schneider’s |  | 4X | 5% | 6.2 μg/ml |  | wing disc, 5 hr |
| Legoff et al., 2013 | Shields and Sang | 2% FCS |  | 2.5% | 125 mU/ml |  | wing disc, 8 hr |
| Handke et al., 2014 | Shields and Sang M3 | 2% FBS | 10X | 5% | 5 μg/ml | 1 ng/ml | wing disc |
| This study | Schneider’s | 2% FBS | 4X |  | 1250 μg/ml |  | discs |
