# Supplementary figures and images for: Long Term Ex Vivo Culture and Live Imaging of Drosophila Larval Imaginal Discs
Source: PLoS One. 2016 Sep 29;11(9):e0163744. doi: 10.1371/journal.pone.0163744 (PMC5042436; doi:10.1371/journal.pone.0163744)

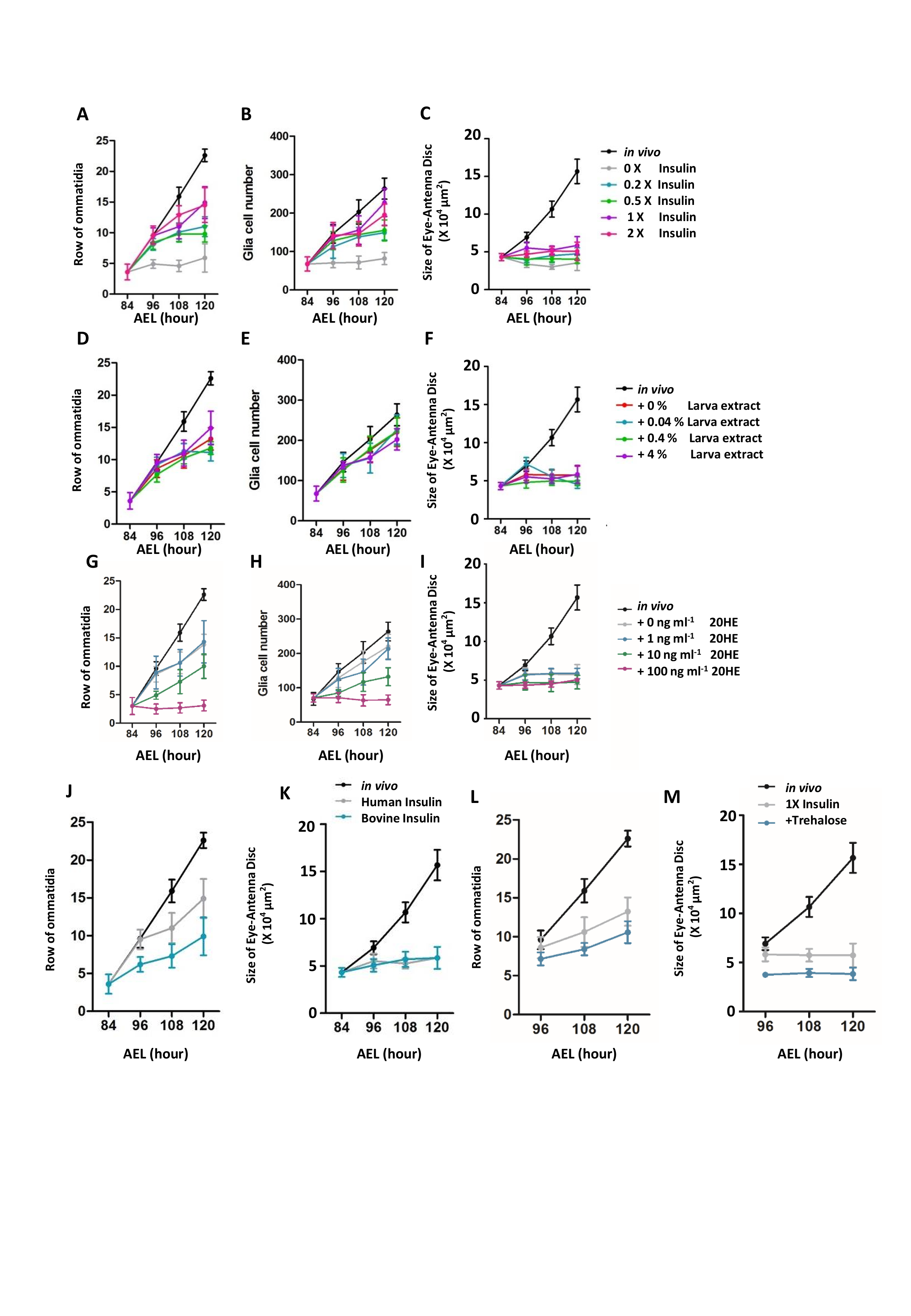

Supplement: S1 Fig — Early third instar (84 hr AEL) w1118 eye-antennal discs were cultured ex vivo for 12 hr, 24 hr and 36 hr (96, 108 and 120 hr AEL), respectively, and compared with discs freshly dissected from larva (in vivo) at these time points. (A-C) Different concentration of insulin were compared. (D-F) The 1X insulin medium with addition of different concentration of larva extract were compared. (G-I) The 1X insulin medium with addition of different concentration of 20HE were compared. (J, K) The insulin from human or bovine were compared. (L, M) The 1X insulin medium with addition of trehalose (60 μg/ml) was compared. (A, D, G, J, L) The number of rows of ommatidia, marked by anti-HRP. (B, E, H) The number of retinal basal glia (marked by anti-Repo). (C, F, I, K, M) the size of eye-antenna disc. In all experiments, N = 20. (TIF) [file pone.0163744.s002.tif]

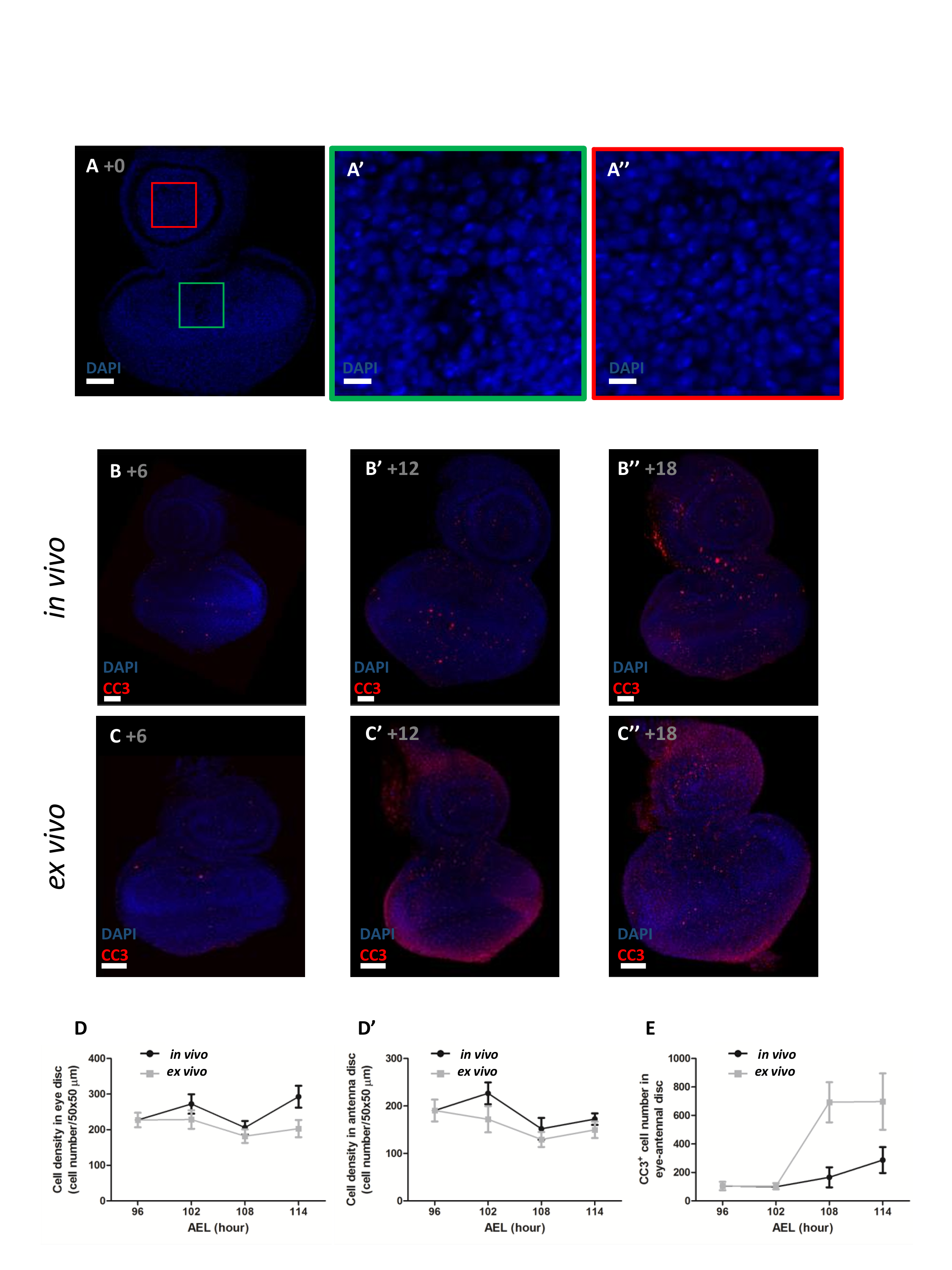

Supplement: S2 Fig — The eye-antenna disc at 96h AEL stained with DAPI (blue). The two insets (green and red, from the antenna and eye disc, respectively) are enlarged as A’ and A”. (B-B”) The in vivo disc at 102h (B, +6), 108h (B’, +12) and 114h (B”, +18) AEL. (C-C”) The ex vivo disc at 102h (C, +6), 108h (C’, +12) and 114h (C”, +18) AEL. The discs were stained with DAPI (blue) and anti-cleaved caspase 3 (CC3, red). The cell density in the in vivo and ex vivo eye disc (D) and antenna disc (D’) were plotted for the 18 hr culture period. (E) The number of CC3+ cells in the in vivo and ex vivo eye-antenna disc were plotted for the 18 hr culture period. The Scale bar is 30 μm, except for A’ and A” (5 μm). (TIF) [file pone.0163744.s003.tif]

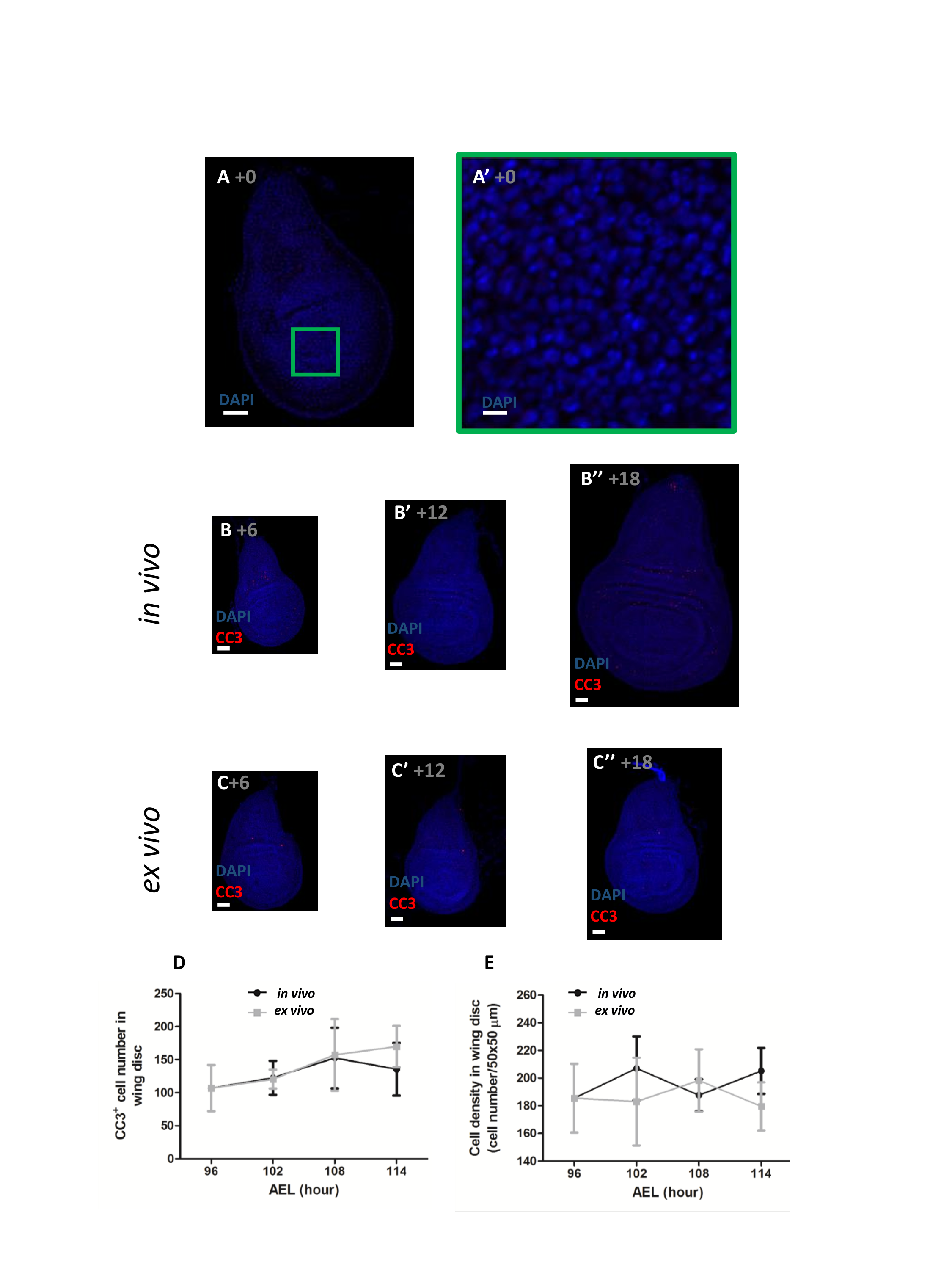

Supplement: S3 Fig — The wing disc at 84h AEL stained with DAPI (blue). The insets (green) is enlarged as A’. (B-B”) The in vivo disc at 96h (B, +6), 108h (B’, +12) and 120h (B”, +18) AEL. (C-C”) The ex vivo disc at 96h (C, +6), 108h (C’, +12) and 120h (C”, +18) AEL. The discs were stained with DAPI (blue) and anti-activated caspase 3 (CC3, red). The cell density in the in vivo and ex vivo wing disc (D) were plotted for the 18 hr culture period. (E) The number of CC3+ cells in the in vivo and ex vivo wing disc were plotted for the 18 hr culture period. The Scale bar is 30 μm, except for A’ (5 μm). (TIF) [file pone.0163744.s004.tif]
